# Linking the Endoplasmic Reticulum to Parkinson’s Disease and Alpha-Synucleinopathy

**DOI:** 10.3389/fnins.2019.00560

**Published:** 2019-05-29

**Authors:** Emanuela Colla

**Affiliations:** Bio@SNS Laboratory, Scuola Normale Superiore, Pisa, Italy

**Keywords:** alpha-synuclein, ER stress, UPR, misfolded proteins, Parkinson’s disease, alpha-synucleinopathy, alpha-synuclein aggregates

## Abstract

Accumulation of misfolded proteins is a central paradigm in neurodegeneration. Because of the key role of the endoplasmic reticulum (ER) in regulating protein homeostasis, in the last decade multiple reports implicated this organelle in the progression of Parkinson’s Disease (PD) and other neurodegenerative illnesses. In PD, dopaminergic neuron loss or more broadly neurodegeneration has been improved by overexpression of genes involved in the ER stress response. In addition, toxic alpha-synuclein (αS), the main constituent of proteinaceous aggregates found in tissue samples of PD patients, has been shown to cause ER stress by altering intracellular protein traffic, synaptic vesicles transport, and Ca^2+^ homeostasis. In this review, we will be summarizing evidence correlating impaired ER functionality to PD pathogenesis, focusing our attention on how toxic, aggregated αS can promote ER stress and cell death.

## Introduction

Misfolded proteins are a common cellular abnormality that is shared among neurodegenerative diseases. Parkinson’s Disease (PD), a multifactorial neurodegenerative disorder that affects the motor, cognitive and peripheral system, is characterized by the accumulation of misfolded, aggregated alpha-synuclein (αS) fibrils into proteinaceous intracellular inclusions in neuronal soma or neurites, named Lewy bodies (LB) or Lewy neurites (LN) (Goedert et al., 2013). The aggregation process of αS is a nucleation-type of reaction where αS monomer converts into a β-sheet conformation that elongates into filamentous structures called protofibrils and fibrils, becoming progressively insoluble (Lashuel et al., 2002; Cremades et al., 2012; Tuttle et al., 2016). The presence of αS inclusions is associated with neuronal damage and has been found in other types of neurodegenerative diseases besides PD that are collectively referred as α-synucleinpathies. In PD one of the most affected neuronal populations, but surely not the only one, is represented by the dopaminergic neurons of the substantia nigra pars compacta. Here widespread dopaminergic neuronal death causes depletion of striatal dopamine, whose reduction is responsible for motor and cognitive dysfunction experienced by PD patients. Since its discovery in 1997, many observations have pointed to the aggregation of αS as one of the culprits of neuronal demise (as examples Feany and Bender, 2000; Masliah, 2000; Lee et al., 2002; Lakso et al., 2003). In addition, the prion-like ability of the protein to spread and propagate its toxic template has shown how αS pathology can disseminate between different anatomically connected areas, from the peripheral nervous system to the brain (Braak et al., 2003; Luk et al., 2012; Masuda-Suzukake et al., 2013; Rey et al., 2013; Holmqvist et al., 2014; Recasens et al., 2014; Sacino et al., 2014). At a neuronal level, αS toxicity has been associated with impairment in numerous cellular functional aspects, including mitochondrial, proteasomal and lysosomal abnormalities, axonal transport deficits and alteration in synaptic transmission (Bendor et al., 2013). More recent evidence has emerged supporting the endoplasmic reticulum (ER) stress, a condition of altered ER functionality, as a mediator of αS toxicity. In this review, we will summarize the link between PD and ER stress, focusing our attention on how pathological αS impairs ER functionality, induces ER stress and ultimately contributes to neuronal death.

## ER Stress and Unfolded Protein Response

Folding of secreted and transmembrane proteins is one of the main functions of the ER. Membrane and extracellularly targeted proteins are translated on ribosomes localized on the cytosolic surface of the rough ER and promptly inserted into the ER membrane or lumen (Görlach et al., 2006). In the ER, proteins achieve a specific folded conformation, acquire post-translational modifications such as glycosylation and formation of disulfide bonds, and are selectively targeted for secretion or destined for the plasma membrane or other cellular compartments. The ER is also responsible for biosynthesis of lipids and steroid hormones and is a primary site for Ca^2+^ storage. Proteins that failed to fold properly are retro-translocated into the cytosol by the ER-associated degradation (ERAD) pathway and degraded by the proteasomes (Smith et al., 2011). To sustain extensive protein folding capability, cells must promptly maintain an adequate level of ER folding machinery and ERAD proteins. Because of the high concentration of proteins in the ER (estimated at 100 mg/mL), the ER quality control is a fundamental mechanism that maintains and preserves cell metabolism and normal functions. Perturbations of this balance lead to accumulation of aberrant proteins in the ER, a condition called ER stress, that if left unchecked, can have deleterious consequences and can lead to the collapse of the whole secretory pathway and cellular homeostasis. In addition to defects in the protein folding machinery, other conditions culminating directly or indirectly in the accumulation of misfolded proteins (including starvation, infections, changes in ER Ca^2+^ concentration and dysregulation in the redox potential of the ER) are able to trigger ER stress. Because of this fundamental role in protein homeostasis, eukaryotic organisms have developed a concerted and coordinated multi-signaling pathway, named unfolded protein response (UPR), that aims to restore ER functionality through the increase of cellular folding capacity and the transient reduction of the flux of proteins entering the ER (Walter and Ron, 2011). To achieve such a status, a massive transcriptional upregulation of ER chaperons, foldases, glycosylases, ERAD proteins, lipid biosynthesis to facilitate ER membrane expansion and, at the same time, degradation of selective mRNA messengers with attenuation of general protein translation, must be well coordinated in order to protect cells from ER stress and recover ER protein quality control (Harding et al., 1999; Hollien and Weissman, 2006; Hollien et al., 2009). However, when the ER stress is too severe and there is a persistent build-up of misfolded proteins that cannot be efficiently eliminated, the UPR can become cytotoxic and can directly initiate programmed cell death through both caspase-dependent and independent pathways (Lin et al., 2007).

**FIGURE 1:**
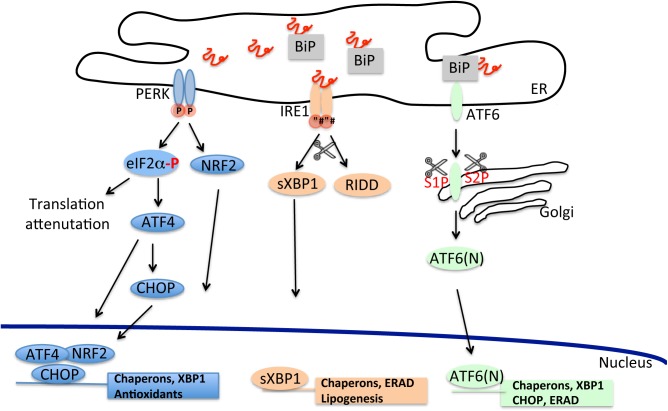
The unfolded protein response cascade. Stressful conditions due to starvation, infections, oxidative damage, and changes in ER Ca^2+^ concentration can lead to accumulation of misfolded proteins in the ER. Induction of the unfolded proteins response through the activation of its three independent arms (PERK, IRE1, and ATF6) counteracts the build-up of misfolded proteins and improves the ER folding capacity.

In eukaryotes, the UPR is highly conserved and comprises three parallel branches, each of them initiated by a specific ER stress sensor. Such sensor is represented by an ER resident protein, which is sensitive to ER perturbations and signals this information to the cytosol and the nucleus. There are three ER stress sensors: (1) the inositol-requiring enzyme 1 (IRE1); (2) the double-stranded RNA-activated protein kinase (PKR)-like ER kinase (PERK); (3) the activating transcription factor-6 (ATF6) (Figure 1).

PERK is an ER-resident type I transmembrane protein with a cytosolic kinase domain. Upon ER stress, PERK phosphorylates the α subunit of the eukaryotic translational initiation factor 2α (eIF2α) at residue Ser51 (Shi et al., 1998). Phosphorylation inactivates eIF2, disrupting the formation of GTP⋅eIF2α⋅Met-tRNAi ternary complex required for mRNA translation leading to a reduction in general protein synthesis and consequently to a decrease of the protein influx into the ER lumen (Harding et al., 2000a; Scheuner et al., 2001). However, in conditions of limited availability of eIF2, specific mRNAs, that contain inhibitory upstream open reading frame sequences in their 5′-untranslated region, are preferentially translated (a process called attenuation). One of these transcripts encodes for the activating transcription factor 4 (ATF4), that selectively upregulates transcription of genes involved in restoring ER functionality such as enzymes for amino acid biosynthesis and transport, protein folding and anti-oxidant response (Vattem and Wek, 2004; Starck et al., 2016). Within ATF4′s known targets is CHOP, a C/EBP homologous transcription factor that controls the upregulation of components involved in apoptosis (Harding et al., 2000a; Ma et al., 2002). Additionally, CHOP binds and promotes transcription of growth arrest and DNA damage-inducible protein 34 (GADD34), an inducible regulatory subunit of the protein phosphatase PP1C. PP1C dephosphorylates eIF2α, providing a feedback mechanism for tightly regulating the phosphorylation status of eIF2α and, in turn, for controlling inhibition of protein synthesis (Connor et al., 2001; Novoa et al., 2001). Another target of PERK kinase activity is NRF2, a transcription factor that induces the translation of antioxidant proteins and detoxifying enzymes (Cullinan and Diehl, 2004, 2006; Marciniak, 2004).

Similar to PERK, IRE1 is a bifunctional ER type I transmembrane protein, highly conserved through evolution and with a carboxy-terminal cytoplasmic kinase and RNase domains (Wang et al., 1998). Mammalian IRE1 has two homologs, IRE1α, an ubiquitous protein and IRE1β which is expressed specifically in the gastrointestinal and respiratory tracts (Bertolotti et al., 2001; Tsuru et al., 2013). In the presence of ER stress, both IRE1 homologs form homo-oligomers through the association of their ER luminal domain. The oligomer then cleaves a 26-base intron from the mRNA encoding the X-box binding protein-1 (XBP1) (Yoshida et al., 2001). Spliced Xbp1, sXBP1, activates downstream a wide set of genes encoding proteins involved in ER membrane biogenesis, protein folding and ERAD (Lee et al., 2003; Acosta-Alvear et al., 2007). In addition to XBP1 cleavage, IRE1 is also implicated in the degradation of specific mRNAs of membrane and secreted proteins, 28S ribosomal RNA and microRNAs as a part of a Regulated IRE1-Dependent Decay (RIDD) pathway (Hollien and Weissman, 2006). RIDD may indirectly contribute to the reduction of protein influx in the ER. However, exhaustive RIDD activity, such as in conditions of chronic ER stress, can indiscreetly degrade messengers of protein involved in protein folding, exacerbating the overload of misfolded polypeptides and indirectly contributing to induction of cell death (Han et al., 2009; Upton et al., 2012).

Unlike PERK and IRE1, ATF6 is an ER-associated type 2 transmembrane protein with (carboxy-terminal luminal domain stress-sensing) a basic leucine zipper domain functioning as transcription factor (Haze et al., 1999). In unstressed conditions, ATF6 is an oligomer that upon activation dissociates into a monomeric form and translocates in the Golgi where it is sequentially cleaved by serine protease site-1 (S1P) and metalloprotease site-2 (S2P) to release an active cytosolic form, ATF6 (N) (Schindler and Schekman, 2009). After migration in the nucleus, ATF6 (N) binds to ER stress response element (ERSE) and activates the transcription of genes involved in ERAD, ER homeostasis and folding machinery such as the ER chaperons BiP/grp78 and Grp94 (Yoshida et al., 2000). Cross-talk between the different UPR pathways has been shown for sXBP1 mRNA whose expression can be unregulated also by PERK and ATF6 (Yoshida et al., 2001; Tsuru et al., 2016) and in the case of CHOP, whose expression appears to be stimulated also by ATF6 (N) (Yoshida et al., 2000; Tsuru et al., 2016).

How can PERK, IRE1, and ATF6 sense misfolded proteins? It is thought that each UPR sensor is maintained in an inactive or quiescent state through binding with the ER chaperon BiP/grp78 (Bertolotti et al., 2000). BiP/grp78 is part of the ER translocon pore and is involved in numerous ER-related functions, such as translocation of nascent polypeptides, protein folding, targeting of misfolded proteins to ERAD machinery and ER calcium homeostasis (Hendershot, 2004). During ER stress BiP/grp78 senses and binds misfolded proteins, dissociating from the binding to the luminal domain of UPR sensors, with concomitant activation of these signaling proteins and initiation of the three different UPR cascades. More recent evidence has suggested that IRE1 can directly sense and bind misfolded polypeptides, without the mediation of BiP/grp78, becoming activated and inducing UPR (Credle et al., 2005; Kimata et al., 2007; Gardner and Walter, 2011). It is not clear whether a similar mechanism applies also to PERK or ATF6 although selective deletion of BiP/grp78 binding site on ATF6 does not result in the constitutive activation of ATF6-dependent branch of the UPR in unstressed conditions (Shen et al., 2005).

Besides its protective function, the UPR has been recently implicated in memory and synaptic plasticity as it has been shown that the PERK-eIF2α branch or XBP1 can regulate gene expression of proteins implicated in long-term potentiation, memory formation and synapsis remodeling (Trinh and Klann, 2013; Martínez et al., 2016). Thus it appears that the UPR is not only a rescue mechanism in case of stressful conditions in the ER but also a way to modulate normal cellular function and homeostasis.

### ER Stress-Induced Apoptosis

When the initial cellular response fails to restore ER homeostasis and misfolded proteins overload cannot be efficiently removed, the UPR switches from an adaptive response to induce cell death. Although the mechanism and key players have not been entirely identified, it appears that the same UPR branches involved in the initial prosurvival response have the capacity to induce apoptosis in the case of severe ER stress. Activation of CHOP by PERK/ATF4 or ATF6 or XBP1 (Harding et al., 2000a,b; Scheuner et al., 2001) appears to be central for the induction of ER stress-driven apoptotic signal. Pro-apoptotic activity of CHOP is mediated by both the upregulation of Bim, a protein that belongs to the BH3-only family (Puthalakath et al., 2007) and by the suppression of the anti-apoptotic protein Bcl-2. The BH-3 only family is comprised of proteins able to induce formation of the mitochondrial pore and, consequently, to induce the release of cytochrome c. Instead, the Bcl-2 protein family inhibits the formation of the mitochondrial outer membrane pore. For CHOP to activate the apoptotic cascade, both factors, Bim and Bcl-2, have to be regulated, although in the opposite direction. Release of cytochrome c then activates caspase-9 in the apoptosome with consecutive cleavage of caspase 3 and initiation of the apoptotic process. More recently, CHOP activity has been proposed to be mediated by PUMA, a p53-upregulated modulator of apoptosis (Galehdar et al., 2010; Ghosh et al., 2012).

In addition to CHOP, upon extensive ER stress, IRE1 can also promote apoptosis through binding to the TNF-α receptor-associated factor 2 (TRAF2) and stimulation of apoptosis signal-regulating kinase-1 (ASK1) (Nishitoh et al., 2002). ASK1 in turn, activates JNK that phosphorylates and inhibits antiapoptotic factors Bcl-2 and Bcl-XL. Interestingly, Bax and Bak, which belong to the BH-3 only family, can modulate directly IRE1 activity by relocating to the ER membrane under ER stress conditions (Zong et al., 2003; Hetz et al., 2006; Klee et al., 2009). Moreover, cells lacking Bax and Bak are resistant to apoptosis after treatment with different ER stress stimuli (Scorrano et al., 2003; Buytaert et al., 2006). Thus, exhaustive ER stress can induce cell death through a tight and well controlled cross-talk with the mitochondria.

However, besides induction of apoptosis through the mitochondria, other pathways have been proposed to take part in cellular demise upon UPR activation. Initial observations had suggested how the initiation of the UPR-dependent cell death cascade could be mediated directly by the ER through activation of the ER-resident caspase, caspase 12 (Nakagawa et al., 2000; Yoneda et al., 2001). However, inhibition of caspase 12 expression in MEF cells does not make cells more vulnerable to ER stressors indicating that caspase 12 is not specifically activated in conditions of ER stress (Obeng and Boise, 2005; Saleh et al., 2006). Also caspase 12 in humans does not appear to be functional whereas the pro-inflammatory caspase 4 seems to now be a more suitable candidate in mediating the ER stress-induced apoptosis (Lu et al., 2014). Nevertheless, the PERK–ATF4–CHOP pathway has been shown to promote ER stress-dependent apoptosis bypassing the mitochondria by recruiting cell death receptors such as TRAIL-R1/DR4 and TRAIL-R2/DR5 and their death ligands (Martín-Pérez et al., 2012; Li et al., 2015). Here, activation of such receptors leads to cleavage of pro-caspase 8 that, in turn, can selectively cleave caspase 3. Moreover, activation of the same PERK branch can also lead to autophagy, where transcriptional upregulation of autophagy-related genes such as Atg5, Atg3, Atg7, Atg10, Atg12, Atg16l1, Becn1, p62, and Nbr, is downstream of ATF4 expression (B’chir et al., 2013). In summary, in case of chronic ER dysfunction, it appears that multiple signal pathways involving the ER, mitochondria and the cytosol can contribute to the ER stress-induced cell death.

### ER Stress and Inflammation

The UPR is also actively involved in inducing inflammation through the stimulation of the transcriptional activity of NF-κB and JNK, key mediators of the proinflammatory response. Bacterial infections can induce all three branches of the UPR and activation of the UPR is necessary for production of proinflammatory cytokines (Smith et al., 2013). Stimulation of Toll-like receptors (TLRs), innate immune receptors known to sense pathogen invasion, such as TLR4 and TLR2, specifically activate the IRE-1/XBP-1 branch for production of cytokines such as IL-1β, IL-6, TNF-α and interferon in macrophages (Martinon et al., 2010; Shenderov et al., 2014). TLR4 signaling appears mediated by MyD88 and TRAF6 that interact and activate IRE-1 through ubiquitination and by blocking its inactivation by PP2A phosphatase activity (Qiu et al., 2013). More recently the activity of two other immune receptors, NOD1 and NOD2, known to sense bacterial cell wall molecules, was shown to be mediated by IRE1α activation after *Brucella abortus* or *Chlamydia muridarum* infections (Keestra-Gounder et al., 2016). In addition to IRE1, also the PERK/eIF2/CHOP pathway can mediate TLR4 signaling during inflammation (Afrazi et al., 2014). In conditions of ER stress, attenuation of global mRNA translation, mediated by the PERK/eIF2α phosphorylation, reduces the protein level of IκB, an inhibitory protein that sequesters NF-κB in a quiescent state through binding. Without IκB, NF-κB can migrate into the nucleus and can transcriptionally activate the upregulation of proinflammatory genes (Deng et al., 2004). In addition to PERK, IRE-1α can also stimulate NF-κB activity, through the recruitment of TRAF2 and consequent binding and activation of IκB kinase (IKK) (Hu et al., 2006). Phosphorylation of IκB by IKK signals selective degradation of IκB through the proteasome and promotes activation of NF-κB. BesidesNF-κB, the IRE-1α-TRAF2 complex can also induce inflammation by direct recruitment and activation of the JNK signaling and consecutive recruitment of AP-1 and transcription of proinflammatory genes (Urano et al., 2000). In addition, other mechanisms, such as the production of reactive oxygen species (ROS) in the ER, the level of glutathione and the release of intracellular Ca^2+^ can activate NF-κB signaling inducing inflammation. Production of ROS, in the form of oxygen peroxide, occurs normally in the ER during the catalysis of disulfide bonds formation and it is mediated by two ER-resident proteins PDI and ERO1 (Görlach et al., 2015). Similarly, oxidative stress in the ER is also the result of increased consumption of glutathione, employed as reducing agent of improperly formed disulfide bonds. Thus, an increase in the ER protein load may lead to an overproduction of ROS and, in turn, may initiate an inflammatory response. To control the level of oxidative stress the PERK pathway, through NRF2 and ATF4, induces transcription of antioxidant and oxidant-detoxifying enzymes, including genes involved in regulating cellular level of glutathione (Cullinan and Diehl, 2004). Thus, ER stress through activation of the IRE1 and PERK branches can directly initiate neuronal inflammation, a key process in the pathogenesis of neurodegenerative diseases, providing a direct link between accumulation of misfolded/aggregated protein and pro-inflammatory conditions.

## ER Stress and Pd Pathogenesis

Several reports support the link between ER stress and PD pathogenesis. One of the first of these was obtained in pharmacological neurotoxic models of PD where acute treatment with MPTP, 6-hydroxydopamine (6-OHDA) or rotenone, in cell cultures induced, although at different extent, activation of the UPR genes (Ryu et al., 2002; Holtz and O’Malley, 2003). Moreover ablation of CHOP in mice protected dopaminergic neurons against 6-OHDA, indicating that the ER stress response contributes directly to neurodegeneration *in vivo* (Silva et al., 2005). Specific sensitivity of the dopaminergic system to ER stress was also confirmed by more recent evidence and could partly explain how this population is particularly vulnerable to protein misfolding. For instance, inhibition of XBP1 protein expression in the substantia nigra of adult mice triggered chronic ER stress and specific neurodegeneration of dopaminergic neurons, whereas local recovery of XBP1 level through gene therapy increased neuronal survival and reduced striatal denervation after 6-OHDA treatment (Valdes et al., 2014). Similar results were obtained in mice after MPTP administration or in neuroblastoma cell lines treated with MPTP or proteasome inhibitors (Sado et al., 2009). In both cases, overexpression of XBP1 rescued neuronal cells from dying, indicating that the UPR plays a pivotal role in dopaminergic neuronal survival. In the same way knocking down ATF6 expression in mice exacerbated neurotoxicity after MPTP insult (Egawa et al., 2011). Interestingly, treatment with MPTP has been shown to induce UPR by affecting ER Ca^2+^ homeostasis through inhibition of store-operated calcium entry (SOCE), whose activity is fundamental for maintaining ER Ca^2+^ level (Selvaraj et al., 2012). In this context, MPTP would inhibit the expression of transient receptor potential channel 1 (TRPC1), a regulator of SOCE, decreasing Ca^2+^ entry into the cells. Overexpression of TRPC1 protected against MPTP-induced loss of SOCE and UPR, while knocking down the gene in mice increased UPR and cell death of dopaminergic neurons. Thus, at least for MPTP, induction of UPR appears to be directly linked to Ca^2+^ imbalance.

Activation of the ER stress response was also reported in human PD brain. Accumulation of ER chaperons was found in LBs (Conn et al., 2004) while increased PERK/p-eIF2α signaling was demonstrated in dopaminergic neurons of the substantia nigra in post-mortem tissue from PD cases, confirming that PD pathology is intimately associated with activation of ER stress *in vivo* (Hoozemans et al., 2007). Interestingly, at least two protective mechanisms against ER stress have been shown to involve modulation of genes such as Parkin and LRRK2, whose mutated forms have been associated with familiar cases of PD. Parkin, an E3 ubiquitin ligase implicated in the regulation of mitophagy, was found increased after treatment with ER or mitochondria stressors and this increase was mediated directly by ATF4 binding to the parkin promoter (Bouman et al., 2011; Sun et al., 2013). Overexpression of parkin protected cells from ER stress by promoting splicing of XBP-1 and the induction of the UPR prosurvival response (Duplan et al., 2013). Also mutations causing loss of function of LRRK2, a protein involved in maintaining neuronal cellular stability, have shown to abrogate upregulation of BiP/grp78 level after 6OHDA treatment or overexpression of αS, enhancing neuronal death *in vitro* and *in vivo* (Yuan et al., 2011). Thus, additional protective mechanisms may be important in preserving cellular environment from detrimental effects of ER stress whereas alteration in such pathways may contribute to PD progression.

### ER Stress and α-Synucleinopathy

In genetic models of PD obtained by overexpression of αS, the association between ER stress and α-synucleinopathy has been studied extensively. In mammalian cell cultures, mice and yeast, toxicity due to overexpression of human wild-type, A53T mutant or C-terminal truncated αS was correlated with ER stress and activation of the UPR (Smith et al., 2005; Cooper et al., 2006; Bellucci et al., 2011; Colla et al., 2012a; Chung et al., 2013; Heman-Ackah et al., 2017). Interestingly, αS-induced dysfunctional ER and ER-stress activated cell death were both attenuated by treatment with L-DOPA, a dopamine analog and the only known treatment for PD at the moment (Song et al., 2017). In pc12 cells, overexpression of mutant αS induced cellular stress in a time-dependent matter that initiated with oxidative and proteasome damage and later culminated with ER stress and activation of ER-stress dependent cell death program (Smith et al., 2005). Blockage of caspases activity with inhibitors, siRNA or treatment with the ER stress inhibitor salubrinal, protected against A53T αS-induced cell death, indicating that the ER stress mediates αS toxicity and contributes to cellular death (Boyce et al., 2005). In addition, since proteasome and mitochondria deficits appeared before UPR activation, this suggested that onset of the ER stress response was the final protective pathway to contain αS damage before apoptosis had to be initiated.

**FIGURE 2:**
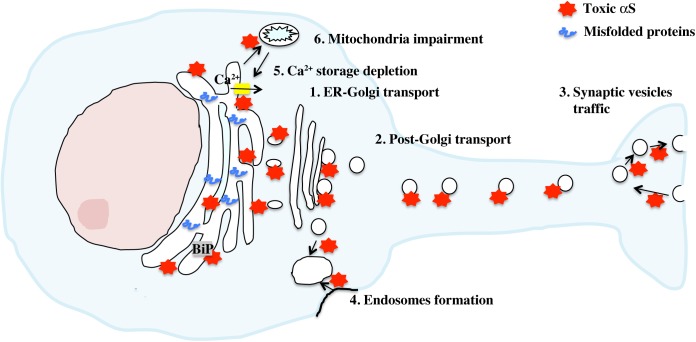
Activation of the UPR by toxic αS. Accumulation of toxic αS in neurons directly affects cellular secretion and proteins traffic at multiple stages thanks to the ability of αS to bind synaptic vesicles and biological membranes. This traffic defect appears to be transferred progressively from the synapse to the Golgi/ER compartments, resulting in a toxic build up of misfolded proteins with concomitant activation of the UPR. Depletion of ER Ca^2+^ level, mediated by direct binding of αS aggregates to SERCA, an ER Ca^2+^ channel, or indirectly through mitochondria impairment increases cytosolic Ca^2+^ and exacerbates ER dysfunction.

Because αS was not known to be a resident protein of the ER, questions on how αS induces ER stress remained open until multiple observations placed αS in close proximity or within the ER and showed its direct interaction with ER and vesicular traffic components (Figure 2). In yeast, overexpression of mutant A53T αS caused toxicity through inhibition ER-Golgi vesicular transport that was completely abrogated by the overexpression of Rab1, a member of the Rab/GTPase family important for intracellular protein traffic modulation (Cooper et al., 2006). Interestingly, Rab1 overexpression was able to rescue dopaminergic neurons from toxicity induced by αS overexpression in other PD animal models, such as *Drosophila, Caenorhabditis elegans* and rat primary cultures. In yeast, vesicular transport deficit was consistent with the inhibition of docking and fusion of vesicles to the Golgi membrane and could be also rescued by overexpression of other members of the Rab family, such as Rab3A and Rab8A (Gitler et al., 2008). Rab3A and Rab8A are responsible for promoting vesicles’ transport at other sites such as the presynaptic button and the post-Golgi element. This suggested that αS overexpression caused traffic defects not only at the ER-Golgi step, but also at multiple sites in the secretion pathway, including at the plasma membrane, an observation that fits well with the physiological role of αS in promoting neurotransmission. As a matter of fact, at the synapse, αS has been described to interact with the SNARE complex and to promote vesicles docking and fusion to the membrane in the presynaptic button (Burre et al., 2010; Diao et al., 2013; Wang et al., 2014). *In vivo*, overexpression of wild-type αS in mice significantly inhibited neurotransmission by delaying vesicles recycling and reclustering after endocytosis at the synaptic terminal (Nemani et al., 2010). In *Drosophila*, overexpression of αS induced accumulation of synaptic vesicles with a larger size at the neuromuscular junction, a defect that was rescued by Rab11 (Breda et al., 2015). Interestingly, in the yeast model overexpressing αS, the observed traffic defect due to the build-up of clustered vesicles unable to fuse, initiated at the membrane level and later expanded in a retrograde manner to the Golgi and the ER. Other reports pointed out that αS could impair traffic of specific vesicles cargo, such as COPII vesicles loaded with ATF6 protein or vesicles containing lysosomal-targeted hydrolases moving from the ER to the *Cis*-Golgi (Credle et al., 2015; Mazzulli et al., 2016).

Additionally, other reports suggested how protein traffic deficit was not due to αS expression *per se* but related to an acquired toxic function. For instance, formation of aggregates in axonal terminals of primary cultures after exogenous administration of αS pre-formed fibrils did not cause initially a generalized defect in axonal transport but impaired primarily Rab7 and TrkB receptor–containing endosomes and autophagosomes cycling (Volpicelli-Daley et al., 2014). Consistent with this observation, we demonstrated in A53T αS transgenic mice how α-synucleinopathy, intended as accumulation of αS toxic species along the ER and the secretory pathway, but not the overexpression of the protein, was associated with the induction of UPR and ER stress-induced cell death *in vivo* (Colla et al., 2012a). Very importantly, appearance of ER-associated αS oligomers preceded α-synucleinopathy and ER stress whereas treatment with salubrinal, delayed α-synucleinopathy onset in transgenic mice and in the AA2V-A53T αS rat model and reduced the level of αS oligomers and aggregates associated with the ER, but not the total amount of αS, suggesting that accumulation of ER-associated αS species results in ER stress (Colla et al., 2012b). Additionally, αS species that accumulate along the secretory pathway appear to have specific distinct biochemical properties compared to non-membranous associated αS aggregates and can be extremely neurotoxic (Colla et al., 2018). For instance, while mature microsomes-αS aggregates isolated from diseased A53T mice exogenously added to mouse primary neurons induced endogenous aggregation and cell death, same species but isolated from presymptomatic mice, without overt α-synucleinopathy, were still cytotoxic but with lesser extent and were found unable to propagate. Such differences in behavior suggest some sort of toxic maturation of αS species right at the ER/microsomal membrane, pushing forward the hypothesis that the ER, Golgi and synaptic vesicles membranes may be a key site for αS aggregation and toxicity.

In addition, we and others have shown that αS interacts with Bip/Grp78 in physiological conditions (Bellucci et al., 2011; Colla et al., 2012a) suggesting that in case of αS aggregation, accumulation of αS aggregates along the ER membrane might directly signal distress to the ER through its interaction with BiP/grp78. Interestingly overexpression of BiP/Grp78 in rats or XBP-1 in *C. elegans* has been shown to alleviate ER stress and protect dopaminergic neurons from αS neurotoxicity (Gorbatyuk et al., 2012; Ray et al., 2014). On the other hand, a study in mammalian cells and αS transgenic mice reported that mutant A53T αS induced cell death and UPR by destabilizing ER Ca^2+^ homeostasis. Overexpression of homocysteine-inducible ER stress protein (Herp), a protein that plays a role in maintaining ER Ca^2+^ balance, markedly reduced A53T αS-induced toxicity in mice, whereas knockdown of Herp exacerbated ER stress leading to a significant rise in toxicity (Belal et al., 2012). Similarly, αS aggregates but not the monomer have been shown to bind to and activate SERCA, an ER Ca^2+^ pump, inducing Ca^2+^ release in the cytosol (Betzer et al., 2018). Treatment with CPA, a SERCA inhibitor, normalized Ca^2+^ level and was neuroprotective against αS aggregates toxicity in *C. elegans.* Ultimately, because ER Ca^2+^ level is particularly sensitive to an increase in ROS, including radical species derived from dysfunctional mitochondria, toxic αS could indirectly contribute and exacerbate ER stress by impairing mitochondria metabolism and the respiratory chain (Görlach et al., 2015).

Thus, because of its preference to bind biological membranes that puts αS in direct contact with the ER/Golgi membrane and synaptic vesicles, toxic, aggregated αS is able to promote ER stress by destabilizing Ca^2+^ homeostasis and inhibiting intracellular protein trafficking and vesicles release, affecting the whole secretory pathway and contributing to the build up of misfolded proteins in the ER with consequent impairment in ER functionality.

## Conclusion

In recent years because of its importance in regulating protein homeostasis, the ER has emerged as a central organelle in the pathogenesis of neurodegenerative diseases. Accumulating evidence support a key role of the UPR cascade in PD progression, correlated specifically to dopaminergic neuronal death and αS toxicity. Specifically, it appears that because of αS’s cellular function and its ability to interact with membranes of organelles and vesicles of the secretory pathway and intracellular protein trafficking, toxicity of αS mediated by aggregation might directly or indirectly affect ER functionality and induce ER stress. In addition because ER stress contributes to promote neuroinflammation, a central process in neurodegeneration, regulating the UPR cascade through therapy may become an efficient cellular target that can lower misfolded protein overload as well as improve inflammation. Thus, future genetic or pharmacology-based approaches tackling the UPR in order to recover ER functionality could potentially lead to disease-modifying therapy in PD and possibly other neurodegenerative diseases.

## Author Contributions

EC conceived the idea, wrote, edited, and reviewed the manuscript before submission.

## Conflict of Interest Statement

The author declares that the research was conducted in the absence of any commercial or financial relationships that could be construed as a potential conflict of interest.
